# Cauldrons of Bronze Age nomads reveals 2700 year old yak milk and the deep antiquity of food preparation techniques

**DOI:** 10.1038/s41598-024-60607-4

**Published:** 2024-06-05

**Authors:** Shevan Wilkin, Jamsranjav Bayarsaikhan, Ankhsanaa Ganbold, Ankhbayar Batsuuri, Lochin Ishtseren, Daisuke Nakamura, Gelegdorj Eregzen, Alicia Ventresca-Miller, Bryan K. Miller

**Affiliations:** 1 Department of Archaeology, Max Planck Institute for Geoanthropology, Jena, Germany; 2 Department of Environmental Sciences, University of Basel, Basel, Switzerland; 3 Institute of Evolutionary Medicine, University of Zurich, Zurich, Switzerland; 4 Australian Research Centre for Human Evolution, Griffith University, Brisbane, Australia; 5 National Center for Cultural Heritage, Ulaanbaatar, Mongolia; 6 Institute of Archaeology, Mongolian Academy of Sciences, Ulaanbaatar, Mongolia; 7 Faculty of Liberal Arts, Saitama University, Saitama, Japan; 8 Department of Anthropology, University of Michigan, Ann Arbor, USA; 9 Museum of Anthropological Archaeology (UMMAA), University of Michigan, Ann Arbor, USA; 10 History of Art Department, University of Michigan, Ann Arbor, USA

**Keywords:** Proteomics, Nutrition

## Abstract

Cauldrons, vessels that are simultaneously common and enigmatic, offer insights into past cultural and social traditions. While assumed to possess a special function, what these cauldrons contained is still largely mysterious. These vessels, such as those made from bronze or copper alloys, function as reservoirs for ancient organics through the antibacterial qualities provided by the metal surfaces. Here we show, through protein analysis, that cauldrons from the Final Bronze Age (ca. 2700 BP) were primarily used to collect blood from ruminants, primarily caprines, likely for the production of sausages in a manner similar to contemporary practices in Mongolia’s rural countryside. Our findings present a different function from the recent findings of cooked meat in copper-alloy vessels from the northern Caucasus 2000 years earlier, exposing the diversity in food preparation techniques. Our secondary findings of bovine milk within the cauldron, including peptides specific to *Bos mutus*, pushes back their regional domestication into the Bronze Age.

## Introduction

For decades, ancient metal cauldrons found across the vast Eurasian steppe have captivated researchers’ attention. In particular, Demidenko’s^1^ research on Ural and Volga region cauldrons, Deydier’s^2,3^ and Pan Ling’s^4^ research on Northern Chinese cauldrons, Erdy’s^3^ research on Hun or Xiongnu-type cauldrons, the Japanese Steppe Archeology Society’s^5^ work on bronze cauldrons in broader Eurasian steppe have demonstrated both their wide regional presence and importance in cultural and political traditions. One of the major distribution areas of the Eurasian cauldrons is present-day Mongolia, yet beyond research on Iron Age imperial Xiongnu (ca. 200 BCE–100 CE) cauldrons, information on large metallic vessels from this region is scant. A number of Scythian era bronze cauldrons have been found in Mongolia (Early Iron Age, ca. 900–300 BCE), but very few with solid archaeological context^6^. Rather than being strategically excavated, most ancient cauldrons from the eastern steppe have been recovered by local herders from locations near river banks and mountainous areas, only later to be housed in museums and cultural heritage institutions. Until now, what these large vessels were used for has been purely speculative, and it has been unclear if, and how, they were used in food preparation. New methods, such as ancient proteomics, can provide meaningful information on the materials cooked, stored, or served from these enigmatic vessels.

Metal vessels are far less common in the archaeological record than those made from ceramics, yet they were still produced and utilised by ancient Eurasian populations. Initially, it appears that the earliest cauldrons were made from copper alloys over 5000 years ago by Eneolithic Maikop populations in the northern Caucasus. Throughout the subsequent millennia, on the far western steppe and eastern Europe cauldrons are thought to have been used for the cooking or serving of food or beverages, and from the large size of the vessels, it is suggested that they were used during large feasts^7–10^. Protein analysis of one such vessel has demonstrated the likely cooking of stew through the presence of meat, blood, and milk tissues within a large copper alloy vessel^11^, however, metal cooking and serving wares were likely used for varied functions across time and space.

**Figure 1:**
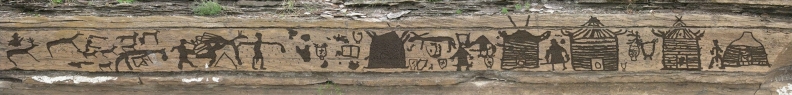
Rock art depiction of Iron Age settlement, Minusinsk, Russia; after Gryaznov 1933^13^. Artwork by Bruce Worden, University of Michigan.

Versions of large bellied cauldrons with a single foot first emerged in the Late Bronze Age (second millennium BCE) in Sintashta cultural contexts of north-central Kazakhstan, and the style then spread mostly across northern areas spanning steppe and forest zones^12^. Cauldrons of the specific type addressed here—of round belly, flanged foot, and two loop handles—occurred throughout northern Eurasia during the Early Iron Age (ca. 900–300 BCE, the so-called Scythian era) and into the era of the Xiongnu Empire (ca. 200 BCE–100 CE). Their significance in communities can be seen in petroglyphs, wherein they are set amid people, animals, and homes, both log cabins and mobile yurts (Fig. 1).

In the Yenesei River regions, near to our site of study, many equivalent cauldrons have been found, some of which, from Sagly Culture tombs in Tuva and northwestern Mongolia, have well-recorded burial contexts that provide insights into their associated foods. These include a ceramic footed-cauldron containing long bones and scapula of sheep/goat, and set next to an assortment of goat skulls, mandibles, ribs, vertebrae, and scapulas (tomb 3, Khairykan; Semenov 2003, 44); and a bronze footed-cauldron with handles set beside sheep vertebrae, scapula and skull, and a goat skull (tomb 33 Chandman/Ulangom^6^).

Similar to non-urban populations in Mongolia today, people in the Late Bronze Age were mobile herders that supplemented their pastoralist diets through hunting and gathering^14,15^. However, archaeological analysis of occupation sites to look at food preparation on the steppe is often complicated by the ephemeral nature of seasonal mobile pastoralist camps that leave little evidence of their presence, as locating them on the landscape is challenging. Compounding this, strong steppe winds deflate the soil, eroding most archaeological deposits from accumulating^16^. Recently, studies of rare complete occupation sites have provided researchers with piecemeal faunal remains, yet these are often either too fragmentary or charred to be identified as domesticated or wild^17,18^.

To date, the sites across the early steppe with the best-preserved skeletal remains, both from humans and animals, are under large stone monuments (kurgans), also referred to as khirigsuurs, slab, or Sagsai burials. Burials underneath these stone monuments are generally well-preserved, and allow for more complete assessment of skeletal remains, including morphological and biomolecular analysis. Previous biomolecular studies of Bronze Age Mongolian populations were heavily reliant on primary and secondary products from terrestrial animals, such as cattle, sheep, goats, and horses, and while they likely consumed a small amount of river resources these were not a primary subsistence resource^15,19–22^. While people were consuming both meat and milk from ruminants and equines in the Bronze Age, which of these products, if any, was present in these vessels has been mysterious.

As this can be investigated through analysis of the residues^11,23,24^, we extracted and analysed proteins from residues held within two Late Bronze Age (2750 BP ± 20) cauldrons from eastern central Mongolia. We hypothesised that each cauldron would contain evidence of tissues from domesticated animals, such as ruminants or equines, as these were commonly herded in the northern regions of Mongolia. Both primary (muscle, blood) and secondary (milk) product use was common during the Bronze Age, providing the potential to identify various types of foods and beverages. Here we present evidence for ruminant blood collection within each cauldron, as well as AMS radiocarbon dating of the leather satchel the cauldrons were wrapped in.

## Materials and methods

**Figure 2:**
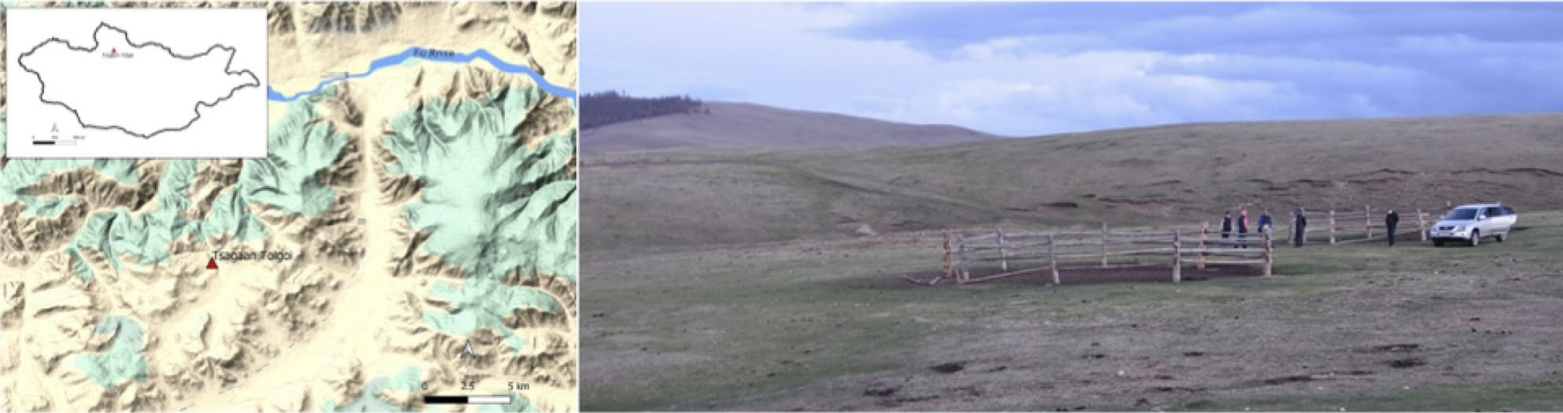
Location of the Tsagaan Tolgoi site. (**a**) Map of the site location in northern Mongolia. (**b**) Image of the landscape at the site from the north (Photo by Jamsranjav Bayarsaikhan).

Both cauldrons, along with a bronze adze, stone mould, broken fragments of bronze objects, and piece of animal leather, were accidentally discovered near the site of Tsagaan Tolgoi by local herders in Tunel Sum, Khuvsgul Aimag in 2021. The find was reported to the National Museum of Mongolia, and the National Centre for Cultural Heritage, and the artefacts were collected in August 2021. The site is located about 9 km to the southwest of the Tunel soum centre, 140 m to the west of the Khoid Khargana stream, at Lat 49.837383 Lon 100.507017 (Fig. 2). There are also several summer camps for the local herders in close proximity to the site.

### Bronze cauldron-1

**Figure 3:**
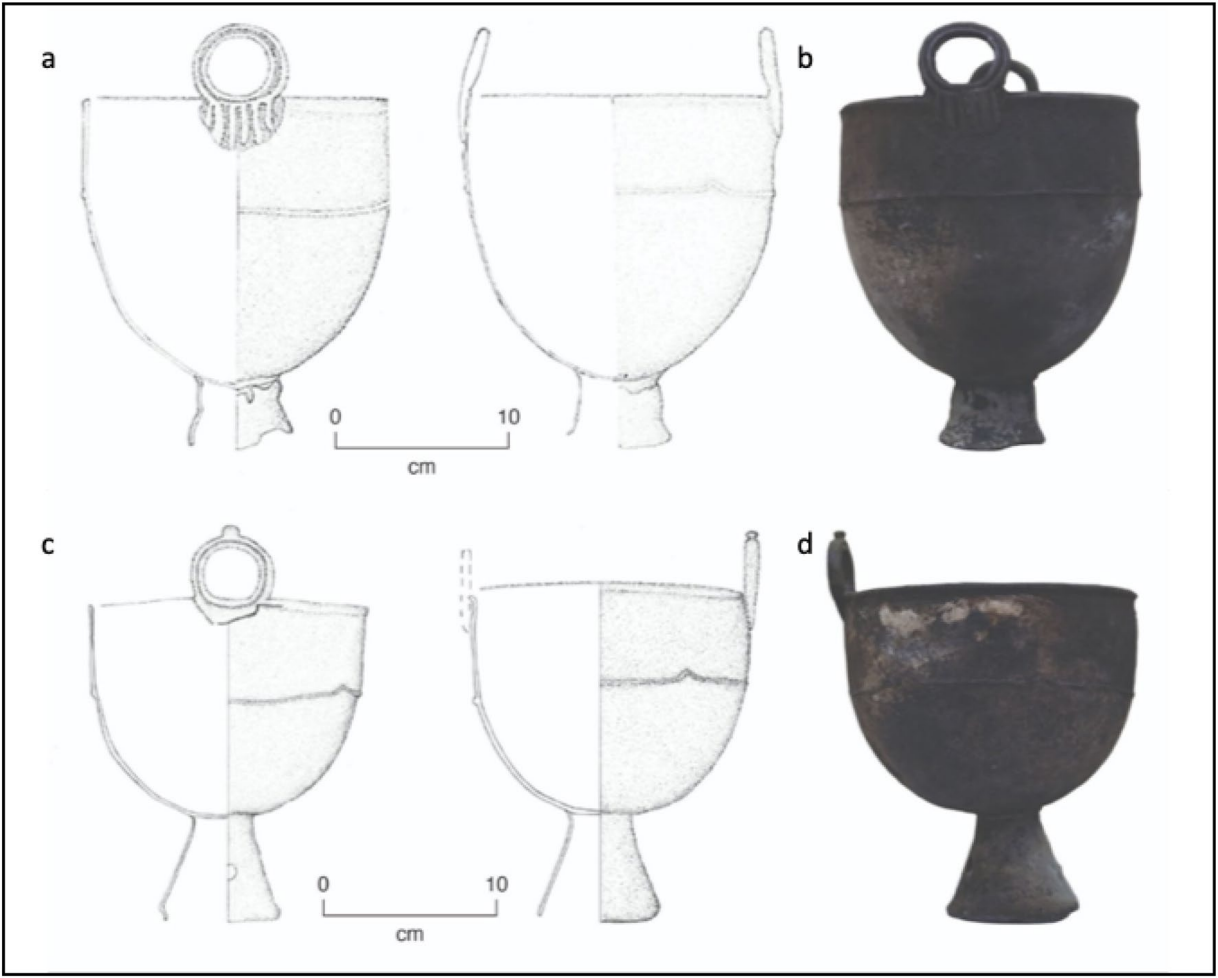
Artistic depictions of what the cauldrons would have looked like during use, and the photographs of the cauldrons at the time of sampling. Bronze cauldron 1 (larger) (**a,b**), Bronze cauldron 2 (smaller) (**c,d**).

The larger of the two cauldrons has a round belly and a pair of ring-shaped handles on the rim as well as a trapezoid shaped base at the bottom. There is soot from burning/heating on both the outside and inside of the vessel. It has a height of 46.2 cm and the diameter across the rim is 36 cm. The round handle has a height of 7.4 cm, with a width of 9.4 cm, the height of the base is 7.5 cm with a diameter of 10.5 × 12 cm (Fig. 3a,b).

### Bronze cauldron-2

The smaller of the two cauldrons has a round belly and pair ring-shaped handles on the rim (one is broken) with a trapezoid shaped base. There is a small hole in the base stand. Soot was also found on both the outside and inside of the vessel. The height of the pot is 38.5 cm, the diameter of the rim is 28.5 cm, the height of the handle is 6.5 cm with a width of 7.8 cm, and the height of the base is 11 cm with a diameter of 12.9 cm (Fig. 3c,d).

## Results

### Radiocarbon date

**Figure 4:**
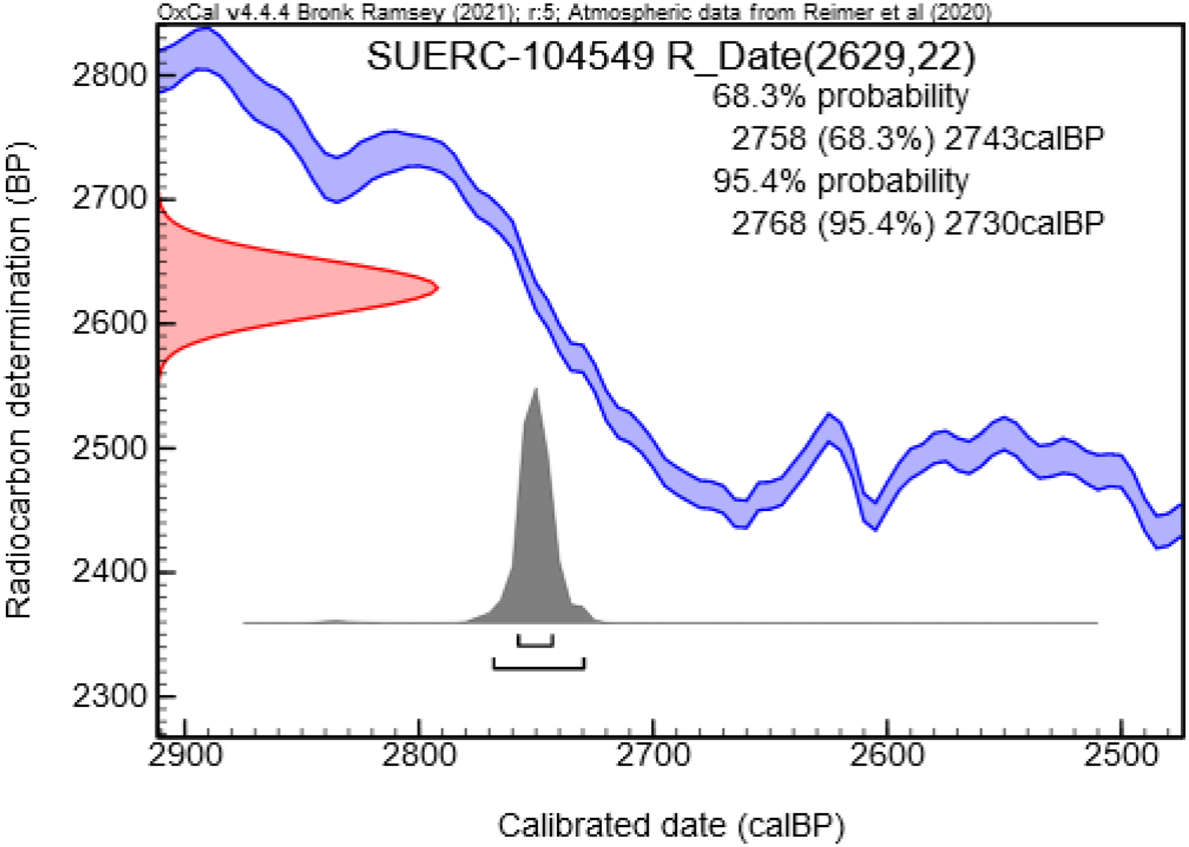
Radiocarbon date range with OxCal calibration.

AMS radiocarbon with OxCal calibration dates show a 95% range from between 2768 and 2760 calBP, with the 68% range with a slightly larger span of 2758–2743 calBP (Fig. 4). This places the sample directly in the Mongolian Final Bronze Age, at the terminal end of the Bronze Age, just prior to the Early Iron Age.

### Proteomic results

#### Large cauldron

**Figure 5:**
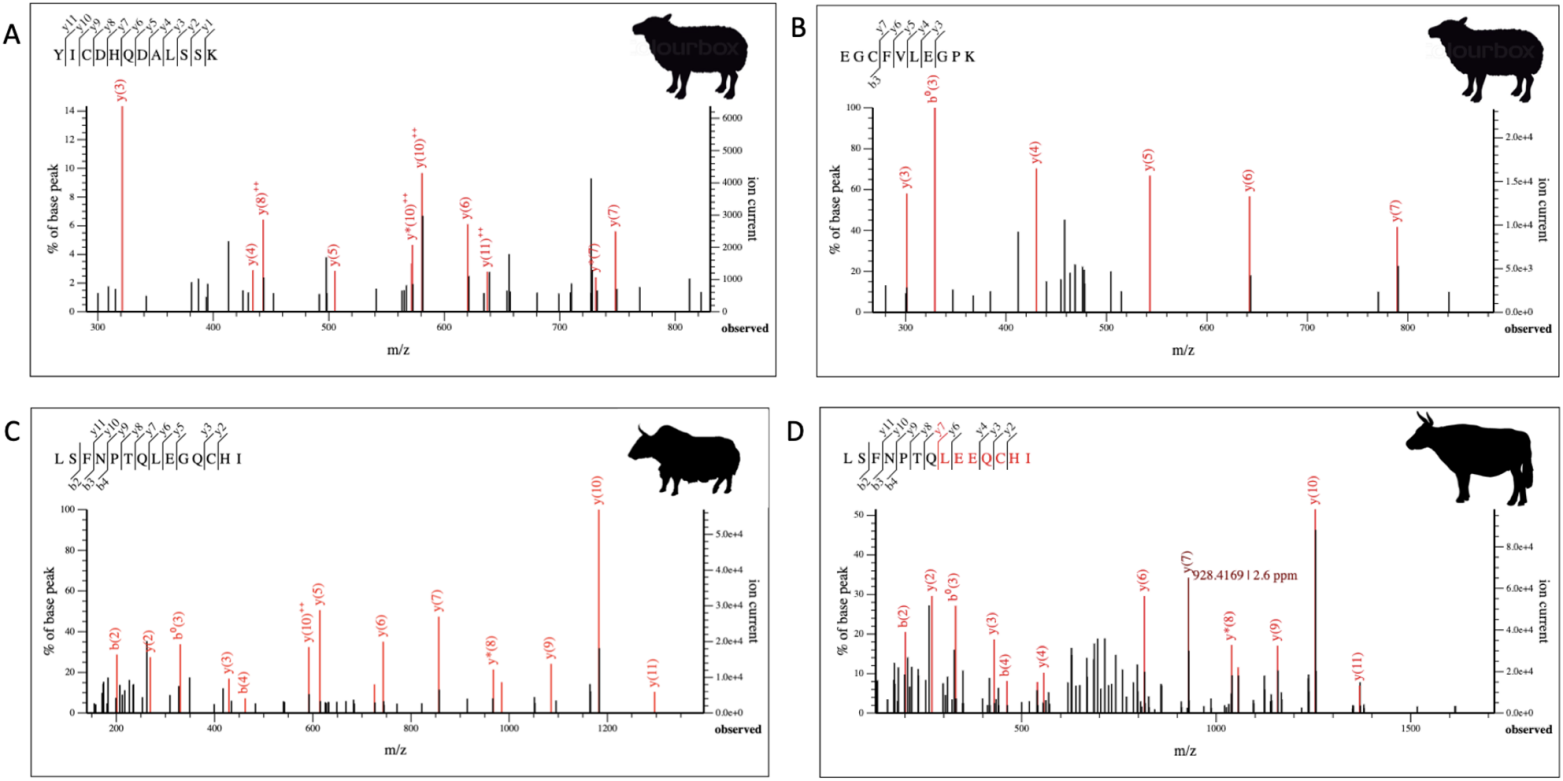
LC–MS/MS spectra for species and genus-specific peptides from both vessels. (**A**) *Ovis* specific albumin peptide from sample Big1A, (**B**) another *Ovis* specific albumin peptide from sample Big1A; (**C**) *Bos mutus* specific (BLG) peptide from sample Small1B; (**D**) Bovinae (*Bos taurus*, *Bos mutus*, and other bovine species) peptide from BLG also from sample Small1B. As yak (*Bos mutus*) has two paralog genes for BLG, they can produce two different peptides.

Blood and blood-related proteins were recovered from the two samples collected from the large cauldron, including: haemoglobin subunit alpha-1, haemoglobin subunit beta, albumin, and alpha-1B-glycoprotein. Both of the hemoglobins are blood proteins, and the glycoprotein, a transport protein, is expressed in the liver and found throughout the body’s bloodstream. Conversely, albumin is found in blood, but has also been recovered from saliva, sweat, skin, and other organs and tissues. Furthermore, bovine serum albumin, while found in one of the large cauldron samples, is a common component of laboratory reagents, and is generally considered a contaminant. In this case, it is impossible to discern whether the albumin peptide spectral matches (PSMs) are from the bovine tissues in the cauldrons or from the laboratory environment. Peptides from each identified protein were classified as belonging to ruminants, with most taxonomic assignments to the subfamily Pecora, the Bovidae family, Caprinae subfamily, and the genus Ovis (Supplementary Table S1) (Fig. 5A,B).

#### Small cauldron

The samples taken from the smaller of the two cauldrons contained blood and milk proteins, as well others with immune-related functions that are found in multiple tissues. Recovered proteins include haemoglobin subunit alpha-1 and 2, haemoglobin subunit beta, albumin, transferrin, beta-lactoglobulin (BLG), alpha-1B-glycoprotein, alpha-2-macroglobulin, complement C3, apolipoprotein A4, cathelicidin-1, proteasome subunit beta, and heme-binding protein 1. Similar to the large cauldron, species identifications were primarily limited to ruminants with most peptides taxonomically specific to the Pecora, Bovidae, and Ovis. However for the milk peptides, there were taxonomic assignments to Bovinae subfamily (cattle, yak, hybrids) (Fig. 5C,D), as well as more specific identifications to *Capra hircus*/*Ammotragus lervia* (goat/Barbary sheep) and *Bos mutus* (yak) (Supplementary Table S1).

## Discussion

Our analysis revealed four peptides from a milk whey protein, BLG, in one of the samples taken from the smaller of the two cauldrons. This is not wholly unexpected, as milking of ruminants was common during the Late Bronze Age in Mongolia, and BLG is the most commonly recovered milk protein from archaeological contexts. However, it remains unclear why these were recovered from a vessel that otherwise seemed to have been used primarily for blood collection. It is possible, and even likely that milk, or a processed milk product, was either purposefully or accidentally incorporated into the vessel during the blood collection, cooking, or processing.

Interestingly, these milk peptides may or may not derive from the same species. One peptide is from the infraorder Pecora, which includes all even toed ruminants (cow, yak, sheep, goat, reindeer, deer) while another is a taxonomically ambiguous sequence that could derive from either Ovis or Bovinae (cow, yak, other cattle) species^20^. The other two BLG peptides align with Bovinae species, with one specific to yak (*Bos mutus*), and the other is specific to the Bovinae subfamily, but could derive from cow, yak, or cattle-yak hybrids. With this combination, all peptides could come from yak or yak/cow hybrids, or they could derive from more than one bovine species. Additionally, one peptide could be from a sheep, and the other could be from any member of the Pecora order. So while all peptides could come from one type of animal, they have the potential to have come from up to four different ruminant species.

**Figure 6:**
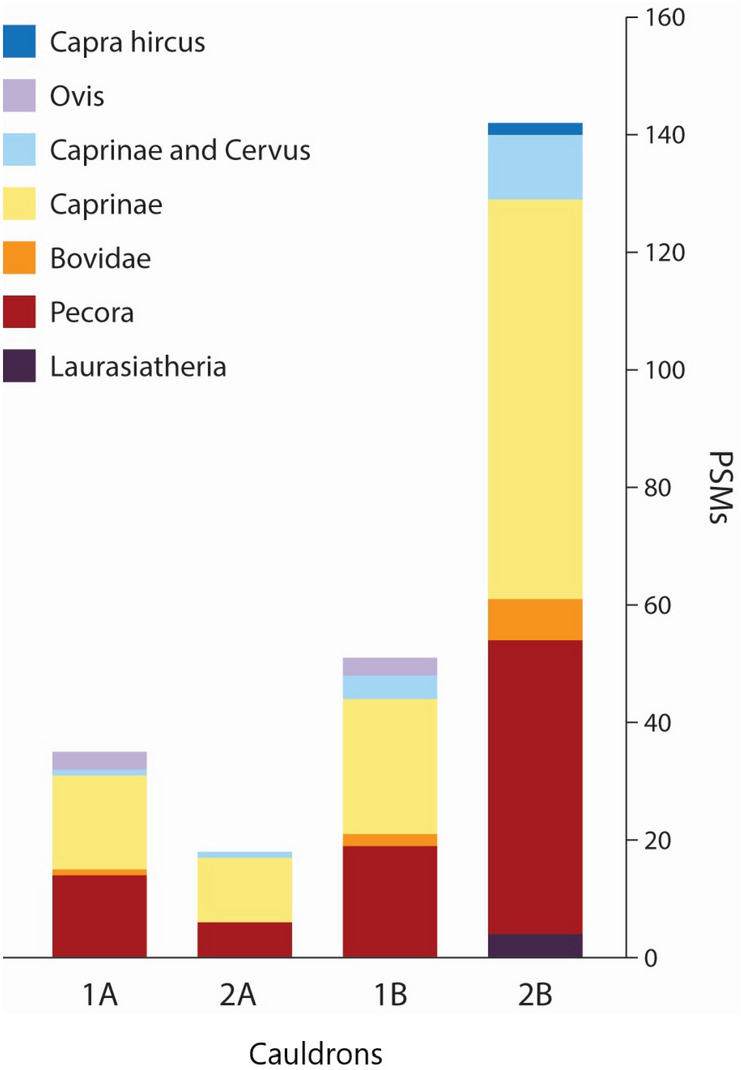
Bar chart of blood proteins recovered from each sample. Numbers along the Y-axis represent the PSMs per sample (Supplementary Table S2). Milk proteins are not included in this figure.

The primary findings of blood and blood-related proteins within both samples from each cauldron clearly demonstrate the collection of ruminant blood from multiple species (Fig. 6). While it is possible that blood could have been collected for raw consumption or ritual purposes, we believe that it is more likely an aspect of food preparation. The use of these vessels as containers for collecting and holding blood aligns well with how blood sausages are made in modern food processing. Today, after the slaughtering of livestock, their blood is collected in plastic or metal bowls, other ingredients are added to the blood, including chopped liver or vegetables, and this mixture is poured into intestines that act as casing for the blood sausage. The species from which the blood derived is consistent with the animal species herded and kept in Northern Mongolia during the Late Bronze Age, as ruminants were widely kept as parts of large pastoral herds.

There are only a couple of written references to blood consumption among Eastern Steppe groups in the Iron Age, which describe Xiongnu customs of “eating meat and drinking blood”^25,26^. It is a clear reference to subsistence, but in the mindset of the Chinese writers who composed such accounts, to eat raw meat or drink blood was commensurate with primitive behaviour. Rather than true accounts, these statements seem like exaggerated portrayals of steppe peoples as barbarians and “others”. There are no archaeological indications of the kinds of meats and bloods consumed, or the ways in which these were described to have been prepared, beyond a few historic references to slaughter techniques common during the Mongol Empire^27–30^, over about 2000 years after our cauldrons were used. When considering both historical and archaeological sources, there has been no reason to give fresh blood consumption in ancient Mongolia serious consideration, but rather that a preference to not waste resources from animal sources^30^ that appears to have extended much further into the past than previously known.

The possibility of blood from both domestic (cow, sheep, goat) and wild (deer) animals is also interesting, as it is common in the past and present for ruminant herding communities to cull members of their herds while also hunting wild game as a part of supplementary subsistence strategies. From the data collected in this study, we cannot unequivocally determine whether wild deer (Cervus) were included in the blood identified in these vessels, however, either the sole collection of blood from domesticated herd animals, or the blood of both domesticated and wild species would be consistent with known subsistence practices. The use of domesticates in subsistence has been well-established through both zooarchaeological and biomolecular evidence^17,18,20,22,31,32^, however, wild deer remains have also been recovered from Mongolian archaeological contexts^17,29^.

Given that horses were commonly part of domestic herds at this time, it was initially surprising that we did not recover any equine proteins, either from blood or milk. However, while horse milk was consumed in Mongolia during the Late Bronze Age, it was not widely found in the Khuvsgul aimag of Northern Mongolia until much later, during the Mongol Empire^21^. In two other studies of milk proteins from dental calculus in the same region of northern Mongolia during the Bronze Age, ruminant milk was ubiquitously recovered, while horse milk proteins were not^19,20^, leaving the absence of equine proteins in our samples less remarkable.

Beyond the intriguing finding of Late Bronze Age yak milk, the findings of other species from our study fall in line with those previously demonstrated to have been present in the same time and space through both zooarchaeological and biomolecular studies^17,19,20,31^. But the use of domestic yak on the Eurasian steppe is mysterious. While there have been tentative zooarchaeological identifications of yak, these often remain equivocal without testing with aDNA. Rock carvings of large-horned bovids in caravans found throughout the Altai region have been deemed as evidence of domestic yaks during the Bronze Age^33^. But these artistic renditions lack the more obvious indicators of yaks, such as bushy tails and long draping belly hair, which do appear in later Xiongnu era (ca.200 BCE–100 CE) depictions. It appears that remains of cow and yak may be osteologically indiscernible from each other, much as the Bronze Age rock art depictions of bovids are, demonstrating that DNA or protein analysis may be necessary to differentiate between the two. And while Xiongnu belt ornaments depicting yaks by-and-large appear in the Sayan-Altai and Southern Siberian regions^34^, areas very suitable for yak herding, the timing of yak domestication in Inner Asia remains unresolved.

The finding of yak milk with ancient proteins is rare, and has yet only been previously identified in a single archaeological sample from the same region^21^, however this was a dental calculus sample taken from an individual who lived during the Mongol Empire/Yuan Dynasty 2500 years later. While not purported to be the evidence of the earliest yak milk in Mongolia, it is the first evidence of recovered yak milk in an archaeological sample. As bovine species are very closely related, the amino acid sequences of each species’ milk proteins are almost identical. For example, in BLG, there is only one amino acid that differs between the *Bos taurus* and *Bos mutus* BLG sequences. Complicating this, the gene in yaks is paralogous, and can result in either sequence. In the cauldron sample, two versions of this particular paralogous peptide sequence were recovered, one that matches specifically to yak, and the other that matches to both yak and cow.

Comparatively, earlier cauldrons (dating to 3900–3300 BCE) that were found within a Maykop kurgan burial at the western end of the Eurasian steppe contained very different protein profiles. Cauldrons from the northern Caucasus region were found to contain blood proteins, but at far smaller proportions compared to proteins from muscle/meat. In that case, the recovered proteins suggest the cooking of a stew containing meat (muscle and blood), as well as milk proteins^11^. These varied findings demonstrate the vast differences in uses of metal vessels from one side of the steppe to the other, and reveal the importance of what biomolecular studies can reveal about food preparation strategies.

Through the recovered preserved proteins, our data suggest that the two particular cauldrons from northern Mongolia were used to collect the blood of ruminant animals during slaughter, and were likely an important part of food production. If the blood was collected, as per our suggestion, for sausage production it would extend the antiquity of this practice at least 2700 years into the past. As ruminant dairying has been practised in the region for over 5000 years, this is hardly surprising. Our finding of Bronze Age yak milk is also intriguing, as it provides insights into when yaks may have first been included in Mongolian subsistence. Yaks are still herded in this area of northern Mongolia today, and while our Late Bronze Age data represents the earliest evidence to date, we suggest that they were likely introduced even earlier.

Our study demonstrates that bronze materials, such as these cauldrons, can act as a reservoir for the preservation of proteins, and likely other biomolecules (DNA, lipids), as has been previously shown with copper alloy vessels and other corroding metallic artefacts^11,24,35,36^. When comparing the data from this study to that of the western steppe cauldrons, it is clear that large vessels were used in different ways across the steppe. Further study of other cauldrons, serving/storage containers, or weaponry can be used to identify species of animals and plants utilised for dietary or non-subsistence related purposes. With thousands of bronze vessels curated in museums around the world, these vessels present the amazing potential for research into ancient food processing and feasting practices.

## Methods

### Radiocarbon dating

The above 14C ages are quoted in conventional years BP (before 1950) and require the calibration to the calendar timescale. The error, expressed at the one sigma level of confidence, includes components from the counting statistics on the sample, modern reference standard and the random machine error. Samples with a SUERC code are measured at the Scottish Universities Environmental Research Centre AMS laboratory and should be quoted as such in any reports within the scientific literature. The laboratory GU coding should also be given in parentheses after the SUERC code. Detailed descriptions of the methods employed by the SUERC Radiocarbon Laboratory can be found as detailed by Dunbar et al.^37^.

### Sampling and protein extraction

Two internal residue samples were taken from each cauldron, resulting in a total of four samples. All samples were extracted in a single batch, with the addition of one extraction blank as a negative control in the dedicated Ancient Protein Laboratory at the University of Zürich. Extractions were done according to a single pot, solid phase, sample preparation (SP3) modified for ancient materials^38^. Residues were demineralised for 3 days in 0.5 M EDTA (pH 8). Following demineralisation, samples were reduced, alkylated, and heated at 99 °C for 10 min. Magnetic beads were used to collect, purify, and digest proteins. After digestions, stage tip clean up was conducted. For a full, detailed protocol see 10.17504/protocols.io.bfgrjjv6.

### LC–MS/MS analysis

Mass spectrometry analysis was performed on an Orbitrap Exploris 480 mass spectrometer (Thermo Fisher Scientific) equipped with a Nanospray Flex Ion Source (Thermo Fisher Scientific) and coupled to an M-Class UPLC (Waters). Solvent composition at the two channels was 0.1% formic acid for channel A and 0.1% formic acid, 99.9% acetonitrile for channel B. Column temperature was 50 °C. For each sample 2 µL of peptides were loaded on a commercial nanoEase MZ Symmetry C18 Trap Column (100 Å, 5 µm, 180 µm × 20 mm, Waters) followed by a nanoEase MZ C18 HSS T3 Column (100 Å, 1.8 µm, 75 µm × 250 mm, Waters). The peptides were eluted at a flow rate of 300 nL/min. After a 3 min initial hold at 5% B, a gradient from 5 to 22% B in 90 min and 5 to 35% B in additional 10 min was applied. The column was cleaned after the run by increasing to 95% B and holding 95% B for 10 min prior to re-establishing loading condition for another 10 min.

The mass spectrometer was operated in data-dependent mode (DDA) with a maximum cycle time of 3 s, using Xcalibur, with spray voltage set to 2.2 kV, funnel RF level at 40%, heated capillary temperature at 275 °C, and Advanced Peak Determination (APD) on. Full-scan MS spectra (350–1200 m/z) were acquired at a resolution of 120,000 at 200 m/z after accumulation to a target value of 3,000,000 or for a maximum injection time of 45 ms. Precursors with an intensity above 5000 were selected for MS/MS. Ions were isolated using a quadrupole mass filter with a 1.2 m/z isolation window and fragmented by higher-energy collisional dissociation (HCD) using a normalised collision energy of 30%. HCD spectra were acquired at a resolution of 30,000 and maximum injection time was set to Auto. The automatic gain control (AGC) was set to 100,000 ions. Charge state screening was enabled such that singly, unassigned and charge states higher than six were rejected. Precursor masses previously selected for MS/MS measurement were excluded from further selection for 20 s, and the exclusion window was set at 10 ppm. The samples were acquired using internal lock mass calibration on m/z 371.1012 and 445.1200.

### Protein data analysis

Raw data files were converted to Mascot Generic Files (MGF) to be searched by Mascot (Matrix Science version 2.7.0.1). Sample MGFs were searched against a database consisting of Swissprot combined with a previously published custom curated dairy protein database^20^. MS/MS ion searches were conducted with trypsin as the digestive enzyme. Carbamidomethyl of cysteine was selected as the fixed modification, with the deamidation of asparagine and glutamine, and the oxidation of methionine as variable modifications. Peptide mass tolerance was set at 10 ppm with an allowance for one ^13^C isotopic shift, and fragment mass tolerance was at 0.01 Da. We allowed for up to three missed cleavages, and the instrument type was set to “Q-Exactive”.

Resulting peptide identifications were filtered with a custom and freely available R script, MS-MARGE^39^ that retains only proteins with at least two distinct peptide spectral matches, peptide e-values below 0.01, and calculates protein and peptide false discovery rates (FDR). We aimed for a protein FDR of under 2% and peptide FDR of under 0.5% for each individual sample, and the actual protein and peptide FDR rates are included in Supplementary Table S1.

### Supplementary Information

Supplementary Legends.

Supplementary Table S1.

Supplementary Table S2.

## Data Availability

Protein data (.raw, .mgf, and .mzid) files are available at 10.25345/C5794151F through the data repository MassIVE (massive.ucsd.edu). Downloads of files are freely available at ftp://MSV000093861@massive.ucsd.edu.
