# Supplementary material for: Cauldrons of Bronze Age nomads reveals 2700 year old yak milk and the deep antiquity of food preparation techniques
Source: Sci Rep. 2024 Jun 5;14:11625. doi: 10.1038/s41598-024-60607-4 (PMC11153608; doi:10.1038/s41598-024-60607-4)
Supplement: Supplementary file 1 — Supplementary Legends. [file 41598_2024_60607_MOESM1_ESM.docx]

*Supplementary table 1: Proteins and peptides recovered per sample. Information for each peptide spectral match includes Mascot score, E-value, post-translational modifications, and false discovery rate for both proteins and peptides.*

*Supplementary table 2: Data used to create Figure 6.*
